# Vitamin D Deficiency (VDD) and Benefits of Supplementation in Veterans with IBS-D

**DOI:** 10.3390/diagnostics13172807

**Published:** 2023-08-30

**Authors:** Chandrasekhar Kesavan, Anjali Das, Preeya Goyal, Christian S. Jackson, Donna D. Strong, Richard M. Strong

**Affiliations:** 1 Department of Gastroenterology, VA Loma Linda Healthcare System, Loma Linda, CA 92357, USA; chandrasekhar.kesavan@va.gov (C.K.); adas2@liberty.edu (A.D.); christian.jackson@va.gov (C.S.J.); 2 Department of Medicine, Loma Linda University, Loma Linda, CA 92354, USA; preeyagoyal25@gmail.com (P.G.); donna.strong2@va.gov (D.D.S.); 3 Musculoskeletal Disease Center, VA Loma Linda Healthcare System, Loma Linda, CA 92357, USA; 4 Presbyterian Intercommunity Hospital, Los Angeles, CA 90602, USA

**Keywords:** diarrhea, vitamin D deficiency (VDD), irritable bowel syndrome with diarrhea (IBS-D), irritable bowel syndrome (IBS), Gulf War veterans (GWVs), Gulf War veteran (GWV), gastrointestinal (GI), post-traumatic stress disorder (PTSD), traumatic brain injury (TBI), Body Mass Index (BMI), C-reactive protein (CRP)

## Abstract

Many veterans deployed to Gulf War areas suffer from persistent chronic diarrhea that is disabling and affects their quality of life. The causes for this condition have eluded investigators until recently and recent literature has shed light on the effect of vitamin D on the brain–gut axis. This study focused on determining clinical causes contributing to diarrhea and assessed whether reversing the identified causes, specifically vitamin D deficiency (VDD), could reduce the incidence of diarrhea in Gulf War veterans (GWVs). All patients completed a workup that included serologies (IBD, celiac), routine laboratory tests (CBC, chemistry panels, TSH, T4, CRP), cultures for enteric pathogens (C diff, bacteria, viruses, small intestinal bacterial overgrowth (SIBO)), and upper and lower endoscopies with histology and a trial of cholestyramine to exclude choleretic diarrhea and rifaximin for dysbiosis. A total of 4221 veterans were screened for chronic diarrhea, yielding 105 GWVs, of which 69 GWVs had irritable bowel syndrome with diarrhea (IBS-D). Paired *t*-tests demonstrated that all GWVs had VDD (t-11.62, df68 and sig(2-tailed) 0.0001) (defined as a vitamin D level less than 30 ng/mL with normal ranges of 30–100 ng/mL) but no positive serologies, inflammatory markers, abnormal endoscopies, cultures, or histology to explain their persistent diarrhea. There was no correlation with age, BMI, or inflammation. Some zip codes had a higher frequency of GWVs with VDD, but the number of deployments had no impact. Treatment with vitamin D supplementation (3000–5000 units), given in the morning, based on weight, reduced the number of bowel movements per day (*p* < 0.0001) without causing hypercalcemia. We suggest that VDD is important in the etiology of IBS-D in GWVs and that vitamin D supplementation significantly reduces diarrhea.

## 1. Introduction

Gastrointestinal problems are common during wars [1,2,3]. These problems are likely triggered by war-related stress, environmental causes, climate change, exposure to war-related chemicals, and emotional stress from being away from family members. Studies on women veterans with irritable bowel syndrome have revealed a prevalence of 33.5% with the most frequently reported trauma being sexual assault (defined as military sexual trauma, MST), in 38.9% [4]. Depression and post-traumatic stress disorder PTSD) were also more common in IBS cases than in controls [4]. The Riddle study [5] from a cohort of active duty members from 2001 to 2009 revealed that women comprised slightly more than 25% of participants, with the majority (66.2%) being white, non-Hispanic men. New-onset IBS was identified in 314 participants, with an estimated incidence of 141.39/100,0000 person-years, whereas the incidence of probable IBS was significantly lower, for a total of 61 incident cases. Studies from Iraq and Afghanistan war veterans found that IBS for both sexes was three times more common with PTSD than without PTSD. Recent literature [6,7,8,9,10,11] suggests that PTSD and depression have a biological effect on systemic inflammation and on brain function that can affect pain perception and sensations of visceral stimuli [5]. The two-way communication with the brain and intestine has effects on the microbiome and dysbiosis through multiple pathways [5]. Recent neuropsychiatric studies have shed light on the role of vitamin D in these conditions [6,7,8,9,10,11]. Vitamin D is known for its role in calcium–phosphorus metabolism, but it also exerts pleiotropic functions via calcitriol-mediated vitamin D receptors. This interaction allows multiple other transcription factors to interact and affect the gene expression of key regulatory factors. It is estimated that 200 to 2000 genes are modulated directly or indirectly by vitamin D [9]. The Riddle study outlined that there was an increased risk for the development of IBS with PTSD, depression, anxiety, previous gastroenteritis, and other stressors such as military sexual trauma (MST) [5], but it was conducted only on active-duty veterans. Our study includes only retired veterans and includes the effect of vitamin D replacement on IBS-D.

The common gastrointestinal (GI) problems these GWVs experience are heartburn, constipation, and diarrhea. These problems are prevalent during active duty in combat areas and after returning to civilian life. They are typically prolonged, leading to a poor quality of life. Many GWVs returning from deployment present to clinics with these chronic GI problems [12]. During Operation Iraqi Freedom and Operation Enduring Freedom, 76.8% of soldiers experienced diarrhea with an average of 15.2 episodes per month [13,14,15,16], and up to 60% of U.S. troops experienced at least one episode of acute diarrhea [16,17]. Traveler’s diarrhea is one of the most common causes of acute diarrhea in military personnel, ranging from 13.6% (Brazil) to 54.6% (Kenya), was prevalent during the Gulf War [16,18,19,20], and is a risk factor for IBS. Acute diarrhea is frequently traced to bacterial or viral etiologies, but in many cases, no pathogen is detected. Chronic diarrhea not traced to organic causes is often considered functional IBS-D [21]. Studies have shown that gastrointestinal infections can result in post-infectious disorders such as IBS-D. After acute gastroenteritis, 4–26% of individuals may develop IBS-D [16,17]. Additionally, up to 30% of individuals [5] with IBS-D are considered to have post-infectious IBS-D [19,22].

Global surveys with servicemen have revealed many gastrointestinal complaints in 23–50% of soldiers [23,24]. A study involving veterans deployed to the Persian Gulf area revealed that 63% of these veterans presented with diarrhea of no organic cause and was consistent with IBS-D [25]. Studies have also revealed that military personnel with a history of traveler’s diarrhea are at a higher risk for gastrointestinal complaints such as IBS-D [19]. These findings demonstrate that IBS-D is the most prevalent gastrointestinal complaint among veterans. A recent study [26] into the mechanisms responsible for symptoms of IBS includes rectal evacuation disorders, abnormal transit, visceral hypersensitivity, bile acid diarrhea, sugar intolerances, barrier dysfunction, the microbiome, immune activation, and chemicals released by the latter mechanism. The recognition of basic molecular mechanisms offers opportunities for the development of future treatments [6,7,8,9,10,11]. The evidence-based management of IBS-D has been the subject of many recent publications with diet [27,28] and drugs [28,29,30,31], and these have led to defined dietary and drug regimens for therapy. However, no ideal diet has been found despite the use of a restrictive fermentable oligosaccharides, disaccharides, monosaccharides, and polyols (FODMAP) diet. The pharmacology suggested has also included eluxadoline, rifaximin, alosetron (moderate certainty), loperamide (very low certainty), tricyclic antidepressants, and antispasmodics (low certainty). Finally, based on our literature review above, there are no studies that clarify the management of veterans who have IBS-D with vitamin D replacement. This study is the first to do so.

Gastrointestinal complaints like IBS (heartburn, constipation, diarrhea) represent a significant disease burden in the United States and globally. The prevalence of IBS is estimated to be active in 14% of adults [32], which accounts for $1562 to $7547 in direct costs and $791 to $7737 in indirect costs per patient per year [33]. IBS impacts negatively on quality of life and lowers the overall perception of well-being [34]. From a cohort of 41,175 active-duty veterans deployed from 2001 to 2009, 314 veterans were diagnosed with new-onset cases of IBS [5]. In the United States, 12% of patients seen by primary care providers have IBS [35,36]. In civilian GI clinics [37,38], IBS diagnosis is also prevalent. After a diagnosis is made, clinicians often attempt to manage symptoms without further investigation. A large percentage of these patients have persistent symptoms that are disabling, including severe diarrhea (bowel movements > 6 times a day with incontinence). There is a need to evaluate for other causes of prolonged IBS symptoms and to determine if reversing this cause changes IBS-D symptoms.

Based on our study and the studies of others, it is possible that the veterans who suffer from IBS-D have more severe symptoms than non-veterans in the number of bowel movements and the association with neuropsychiatric diseases such as post-traumatic stress disorder (PTSD), depression, anxiety, and traumatic brain injury (TBI). All are known to increase IBS-D in veterans. In a recent review, ref. [4] outlined animal studies exploring altered microbiomes, leaky gut, and enteric nervous system dysfunction caused by toxic chemical exposure (pyridostigmine bromide). Whether dysbiosis, leaky gut, or chemical exposure causes increased exposure to inflammatory cytokines in humans as in rodents has not been proven. Vitamin D deficiency is a known mediator of the severity of inflammation in autoimmune diseases such as rheumatoid arthritis, psoriasis, and inflammatory bowel disease, and supplementation is the standard of treatment. Low vitamin D levels are also associated with a dysregulated hypothalamic–pituitary–adrenal axis (HPA) [7], but the relationship with neuropsychiatry is just being established. A key factor in the onset and progression of all these neuropsychiatric disorders is neuroinflammation, likely mediated by vitamin D deficiency [9]. Therefore, we tested for vitamin D deficiency (previously defined as <30 ng/mL, normal range 30 ng/mL to 100 ng/mL) in GWVs with chronic diarrhea and determined whether replacement would have a positive effect on this condition.

## 2. Materials and Methods

### 2.1. Patients

Approximately 4221 patients were seen from 9 January 2014 to 9 January 2020 at the VA Loma Linda Healthcare System in the Digestive Care Clinic. Of these patients, there were 105 GWVs with IBS-D. Sixty-nine patients had completed the evaluation and follow-up, which provide the basis for this study. Patients were followed prospectively, and data were analyzed retrospectively. All patients were evaluated and managed by one investigator who attended all the clinics (RMS).

### 2.2. Patient Evaluation

All patients were subjected to the same evaluations, which included upper and lower endoscopies with biopsies, duodenal aspirates for culture, stool calprotectin, cultures for parasites and enteric pathogens, radiographic upper and lower series, computed tomography (CT) scans, and routine laboratory tests for tissue transglutaminase, IgA levels, C-reactive protein (CRP), CBC, thyroid, and chemistry panels. Patients also underwent a trial of cholestyramine for bile-salt diarrhea and rifaximin for small intestinal bacterial overgrowth (SIBO) and dysbiosis. This workup follows the guidance of many organizations specializing in IBS and, most recently, the United European Gastroenterology and European Society for Neurogastroenterology and Motility [39].

### 2.3. Data Collection

Data collection included age, gender, zip code, Gulf War deployments, the number of bowel movements before and after vitamin D treatment, vitamin D doses, levels before and after treatment, and adverse events after replacement.

### 2.4. Statistical Analysis

The data are presented as the mean ± SEM. We used Student’s *t*-test to compare differences between the groups and one-way paired *t*-tests to evaluate vitamin D deficiency within the groups based on established standards. SPSS version 21 was used to perform the statistical analyses. *p* < 0.05 was considered significant between the groups.

## 3. Results

We performed an extensive investigation, previously described, and found only VDD in all these patients. There were no signs of inflammation in histology tests or in the routine chemical markers (CRP, ESR). One paired *t*-test revealed that vitamin D levels were significantly (*p* < 0.01) reduced in GWVs compared to standard normal values (30–100 ng) (Table 1).

**Figure 1:**
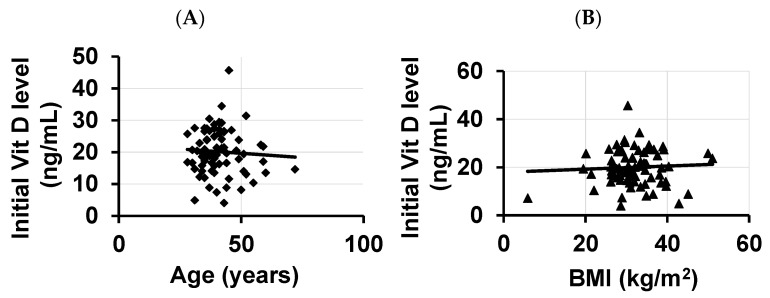
Correlation analysis between initial vitamin D levels with the (**A**) age and (**B**) BMI of the study population.

We assessed whether the vitamin D levels in GWVs were influenced by BMI or aging. We found a non-significant negative correlation (r = −0.05 to −0.06) between age and BMI (Figure 1A,B).

**Figure 2:**
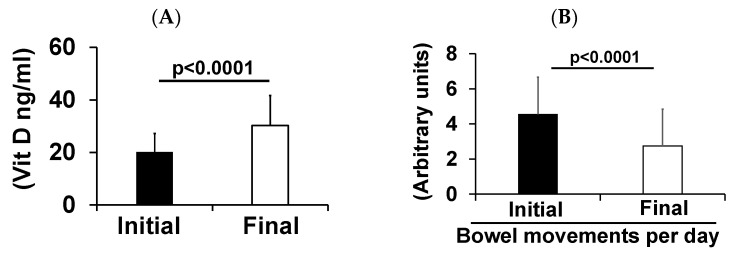
(**A**) Vitamin D levels and (**B**) the number of bowel movements between the initial (before treatment) and final (post-treatment) treatment.

**Figure 3:**
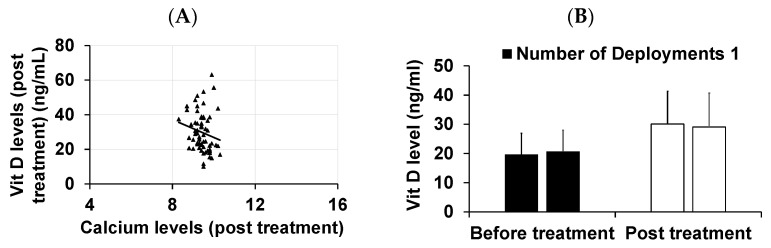
(**A**) Correlation analysis between vitamin D levels and calcium post-treatment and (**B**) vitamin D levels with the number of deployments.

Vitamin D levels were assessed during re-visits. It was found that the vitamin D levels in the GWVs with IBS-D significantly increased after treatment (Figure 2A). The impact of improved vitamin D levels significantly reduced the number of bowel movements (Figure 2B) in our patients. Since vitamin D therapy can increase calcium levels, a correlation analysis between vitamin D and calcium levels was performed, demonstrating a weak negative correlation. This suggests that the dosage utilized did not induce hypercalcemia (Figure 3A). We considered that the number of deployments to Gulf War areas might influence VDD. Our analysis revealed that even one deployment was sufficient to cause vitamin D deficiency (Figure 3B).

## 4. Discussion

In our study, we found that the returning GWVs suffered from severe IBS-D, which had a significant impact on their quality of life. There are several potential causes for chronic IBS-D in the GWVs that we considered investigating, such as poor intestinal absorption, chronic infection causing inflammatory responses, small intestinal bacterial overgrowth (SIBO), changes in the innate immune system, and the use of psychiatric medications to treat traumatic brain injury (TBI), post-traumatic stress disorder (PTSD), depression, and anxiety. Though many patients were already on psychiatric medications for anxiety, PTSD, and depression, their diarrhea persisted. Based on the literature review, we speculated that a hidden chronic infection or a change in the innate immune system might be the most likely cause, but how to diagnose it with routine studies was the enigma.

Vitamin D is essential for the formation of healthy bones and in regulating cellular inflammatory processes directed by inflammatory cytokines [40]. Measuring vitamin D levels using the 25-hydroxyvitamin D concentration (25(OH)D) is still controversial for defining vitamin D status [41], but the involvement of the non-skeletal aspects of vitamin D is not. Vitamin D is known for its role in calcium–phosphorus metabolism, but it also exerts pleiotropic functions via calcitriol-mediated vitamin D receptors. This interaction allows many other transcription factors to interact and affect diverse signaling mechanisms. It is estimated that 200 to 2000 genes are modulated directly or indirectly by vitamin D [9]. A recent study looked at PTSD and reduced vitamin D levels, specifically the role of vitamin D-binding protein and group-specific component (GC) by testing polymorphisms (rs4588 and rs7041) and concluded that altered vitamin D metabolism is involved in PTSD [11]. In a mouse study, vitamin D was shown to protect against traumatic brain injury via modulating TLR4/MyD88/NF-kappa B-signaling pathways, and vitamin D deficiency is associated with poor functional outcomes at hospital discharge and mortality at 6 months after injury in TBI patients with intracranial hemorrhage or diffuse axonal injury [42]. Recent studies on the regulation of immune function with SARS-CoV-2 infection demonstrated how the expression of the enzyme CYP27B1 by the airway epithelium and alveolar macrophages that produce the active metabolite of vitamin D (1,25 (OH)_2_D) and the vitamin D receptor (VCR) that promotes the innate immune response [43]) influences the severity of the viral infection. These studies suggest the complicated interaction of vitamin D and vitamin D receptors and signaling that affects function in many conditions that can affect veterans, including IBS-D, PTSD, depression, and TBI, to name a few. There is a probability that GWVs’ innate immune system is altered, causing an imbalance in the levels of vitamin D metabolic enzymes (CYP24B1 hydroxylase, which increases vitamin D levels, and CYP24A1 hydroxylase, an enzyme that reduces vitamin D levels), resulting in VDD. Further studies are necessary to test this hypothesis.

Numerous studies have shown that increased body weight, sex, genotype, ethnicity (African American), and aging are associated with lower 25 hydroxyvitamin D (25(OH) D) concentrations, but in our study, neither age nor BMI was the cause of decreased vitamin D levels. We were unable to re-affirm ethnicity effects due to the small number of African Americans in our patient population, but Riddle [5] did demonstrate that IBS-D was more common in white non-Hispanic males. Adult studies have shown that VDD is highly prevalent in patients with IBS [44], but the response to replacement has not been published. Studies in children also have shown a high prevalence of VDD [45,46].

Our findings show that VDD is present in our GWV population, and that supplementation improves their symptoms, although not completely. This suggests that VDD might be only one of the causes of IBS-D in this patient population (GWVs). GWVs with VDD were provided with daily oral vitamin D doses, ranging from 3000 to 5000 units, to be taken in the morning. Importantly, since vitamin D is a fat-soluble vitamin, taking it in the morning when the body is physically active should increase the absorption rate and distribution of vitamin D to different body sites. A recent study of vitamin D dosing found that daily supplementation was preferred, and parenteral supplementation was used only for malabsorption states, as was done in this study [47].

We found that vitamin D therapy did not completely resolve the diarrhea in our patients. While other studies have shown that daily vitamin D supplementation did not change stool weight [48], some studies have shown that vitamin D supplementation is superior to placebo for IBS treatment [49] and that VDD is common in IBS patients [50].

Studies have shown that patients with diarrhea have increased expression of cytokines (i.e., IL6) [40,51]. Since VDD increases these pro-inflammatory cytokines (IL6, MCP1) through NF-kB and TNFα mechanisms, and because GWVs are often exposed to war-related stress, burn pit hazards, climate change, war-related chemicals, and military sexual trauma (MST), which are known to induce inflammatory responses, we investigated if diarrhea in GWVs was associated with an increased inflammatory response. We assessed inflammatory responses by the C-reactive protein (CRP) levels before and after vitamin D treatment, surprisingly finding a negative correlation (r = −0.39) between initial vitamin D levels and CRP. Supporting this observation, histology did not demonstrate inflammation. While the causes for IBS-D in GWVs could be different, the vitamin D replacement data do show effectiveness in reducing the number of bowel movements.

A weakness of our study is the lack of women and other ethnic groups. The published literature is similar, showing that white, non-Hispanic males make up the majority of this population. This might suggest a genetic interaction with vitamin D metabolism that could be studied. A better representation of all those veterans in this study with PTSD, depression, anxiety, and MST and the antipsychotic medications they were taking might have been helpful, as this is a complicated population of veterans. Measuring levels of inflammatory cytokines (IL6. MCP1. TNF-alpha), CYP24P1/CYP24A1 hydroxylases, and vitamin D-binding protein polymorphisms (rs4588 and rs7041) might also be helpful in the future. It seems reasonable to consider a trial of steroids (budesonide) or biologics used to treat inflammatory bowel diseases (IBD) in the most refractory cases, considering the possibility of altered cytokines.

## 5. Conclusions

In summary, our findings demonstrate that administering a daily dose of vitamin D in the morning in GWVs suffering from IBS-D can have a significant positive impact on diarrhea frequency and quality of life without complications. Age, BMI, and the number of deployments had no effect on vitamin D levels.

## Figures and Tables

**Table 1:** One paired *t*-test of vitamin D levels against the standard value of vitamin D.

	N	Mean	SD	SEM
Vitamin D initial level	69	20.06	7.10	0.86
	Test value = 30.0			
Vitamin D initial level	*t*	df	Sig. (2-tailed)	
	−11.62	68	0.000	

T(68) = −11.62, *p* = 0.0000.

## Data Availability

The data presented in this study are available.
